# The Effect of Nucleating Agents on Enzyme-Induced Carbonate Precipitation and Corresponding Microscopic Mechanisms

**DOI:** 10.3390/ma15175814

**Published:** 2022-08-23

**Authors:** Yuanjiang Yang, Mingdong Li, Xueqing Tao, Shiai Zhang, Jia He, Liping Zhu, Kejun Wen

**Affiliations:** 1 School of Civil and Architectural Engineering, East China University of Technology, Nanchang 330013, China; 2 College of Civil and Transportation Engineering, Hohai University, No.1, Xikang Road, Nanjing 210098, China; 3 School of Water Resources and Environmental Engineering, East China University of Technology, Nanchang 330013, China; 4 Department of Civil and Environmental Engineering, Jackson State University, Jackson, MS 39217, USA

**Keywords:** plant urease, calcium carbonate, nucleating agent, unconfined compressive strength

## Abstract

Plant urease has the advantages of high activity and small size in enzyme-induced calcium carbonate precipitation (EICP). However, there area lack of nucleation sites for calcium carbonate in EICP. Sucrose and sorbitol, which are readily available and inexpensive, have the potential to provide nucleation sites for EICP as nucleating agents. To explore the effects of the two nucleating agents on EICP, the productivity of calcium carbonate, unconfined compressive strength (UCS) and microscopic mechanisms were tested. It is found that the productivity of EICP can be increased as much as 5.1% by the addition of sorbitol with an optimal content of 5%, and the productivity of EICP can be increased as much as 12.3% by the addition of sucrose with an optimal of 4%. The UCS of EICP-treated sand increases by 2.2 times after being improved by sorbitol with a content of 5.2%, the CaCO_3_ content of EICP-treated sand with sorbitol added increased by 1.5% compared to conventional EICP-treated sand. These results show that the two nucleating agents are effective for improving EICP. The SEM images verify that sorbitol/sucrose can compensate for the lack of nucleating sites in EICP and explicate the effect of nucleating agents on EICP.

## 1. Introduction

Biomediated soil improvement technology has been extensively studied in recent years, and biological cementing through calcium carbonate precipitation has good prospects [1,2,3,4,5,6]. There are three main ways to produce biomediated calcium carbonate precipitation, including urea hydrolysis, sulfate reduction and microbial denitrification [7,8]. Urea hydrolysis-induced calcium carbonate precipitation attracts the widest attention [9,10,11,12] due to the highest efficiency.

**Figure 1:**
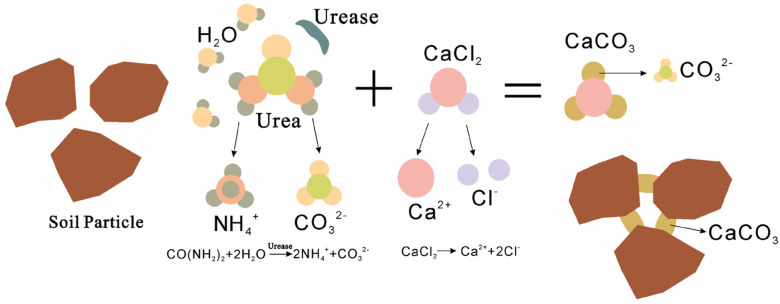
Summary of the carbonate precipitation process through urea hydrolysis.

The improvement process of soil by urea hydrolysis-induced calcium carbonate precipitationis shown in Figure 1. CO_2_ and NH_3_ are generated when urea is hydrolyzed, and NH_3_ is dissolved in water to form hydroxide and NH_4_^+^. Due to these ions, the pH value of solution increase and the CO_2_ dissolved in water reacts to produce CO_3_^2−^. At this time, Ca^2+^ in calcium source combines with CO_3_^2−^ to form calcium carbonate, and calcium carbonate precipitation accumulates at the contact point of soil particles to produce cementing and enhance the strength of soil mass. The reaction speed can be increased 10^14^ times [13] when urease as catalyst is present in the hydrolysis reaction.

(1) $$\mathrm{CO}{\left({\mathrm{NH}}_{2}\right)}_{2}+2H_{2}O\overset{\mathrm{Urease}}{\to }2{\mathrm{NH}}_{3}+{\mathrm{CO}}_{2}↑$$

(2) $$2{\mathrm{NH}}_{3}+{\mathrm{CO}}_{2}+H_{2}O\to 2{\mathrm{NH}}_{4}^{+}+{\mathrm{CO}}_{3}^{2-}$$

(3) $${\mathrm{CaCl}}_{2}\to {\mathrm{Ca}}^{2+}+2{\mathrm{Cl}}^{-}$$

(4) $${\mathrm{Ca}}^{2+}+{\mathrm{CO}}_{3}^{2-}={\mathrm{CaCO}}_{3}↓$$

Urea hydrolysis utilizing bacteria (sporosarcina pasteurii) as a source of urease enzyme for soil cementation was first discussed by Whiffin (2004) and later called “microbial-induced carbonate precipitation (MICP)” [12]. Various studies have examined the potential of MICP in addressing many challenges in granular soils, such as erosion resistance, slope stability, under-seepage of levees, and the bearing capacity of shallow foundations [5,14,15,16]. However, several drawbacks of MICP have limited its field application, such as the relatively large size of microbes for fine-grained soils, confirming its inappropriateness for fine-grained soils [1,17,18]. In addition, since bacteria are living organisms, a suitable environment is required for bacterial growth and enzyme production [6,19,20,21].

The technique of “enzyme-induced carbonate precipitation (EICP)” that uses free urease extracted from plants as the enzyme source has a broad application scope [22,23,24,25,26]. Urease shows relatively high activity within the temperature range of 15–75 °C [27]. Free urease has extremely small size and produces no additional polymeric substances. Thus, the possibility of biological blockage is greatly reduced and the application scope of EICP is extended to finer soils [28,29]. There are great prospects for the biological technology of soil improvement by promoting calcium carbonate precipitation through plant urease.

The catalytic effects of EICP varies for different plant ureases. Jack bean and soybean are used extensively in the current research as the main source of plant urease. The latest research shows that black beans have higher urease activity than soybeans [30], which may become another source of urease in EICP. Unlike MICP, EICP has no nucleation sites that can bridge soil particles. The absence of these nucleation sites may cause an unfavorable influence on the precipitation morphology of the calcium carbonate and as a result, weaken the effect of calcium carbonate cemented soil. The proteins in skim milk combine with Ca^2+^ in EICP solution to form aggregated calcite or precipitate as nucleation points for carbonate precipitation [31]. Almajed et al. argued that adding skim milk into EICP solution could significantly improve the compressive strength of the soils treated with EICP. This increase in strength maybe attributed to the nucleation points formed by the skim milk [32].

Skim milk is not suitable for large-scale use in geotechnical engineering due to its high price, and it is necessary to find some less expensive nucleating agents to replace skim milk. In this paper, the cheaper sucrose and sorbitol are used as nucleating agents to explore their effects on sand improvement by enzyme-induced carbonate precipitation (EICP). In addition, using black soybean and soybean as urease source, the calcium carbonate productivity of enzymatic precipitation in EICP with black soybean urease with higher urease activity and widely used soybean urease was compared. A series of experimental tests were conducted with sucrose and sorbitol as nucleating agents. The calcium carbonate productivity of the modified EICP and the unconfined compressive strength of the treated sand were tested. The effects of nucleating agents on the shape, crystal form and distribution of calcium carbonate in sand particles were studied by XRD and SEM.

## 2. Materials and Methods

### 2.1. Materials

**Figure 2:**
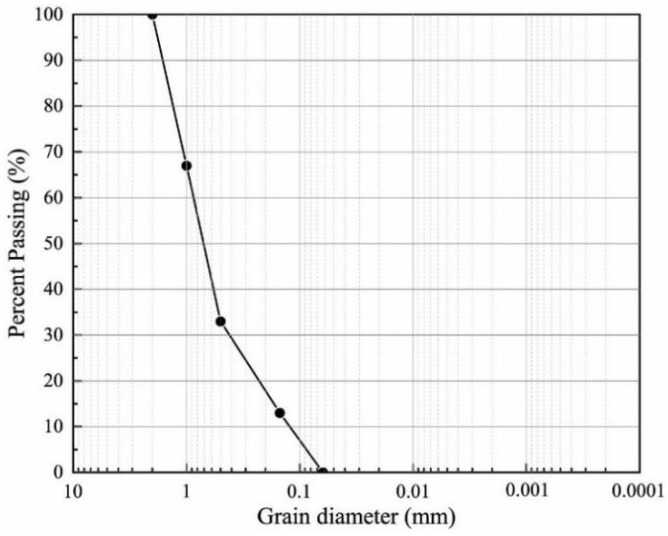
Grain size distribution curve.

ISO standard sand was selected as raw material to be improved in this test, whose particle size distribution curve is shown in Figure 2.

Soybeans and black beans purchased in the market were used as urease sources. The primary materials, such as urea, CaCl_2_, sucrose and sorbitol were obtained from Tianjin Zhiyuan Chemical Reagent Co. LTD., Tianjin, China. The cementing solution was a mixture of urea with a purity of 99% urea and CaCl_2_ with a purity of 96%.

### 2.2. Methods

#### 2.2.1. Extraction of Urease

The soybeans and black beans were crushed into powder in the mill. The fine powder was sieved out with a 0.25 mm sieve and stored in a low temperature dry environment. Then, 40 g/L of soybean powder solution was prepared by adding the sieved soybean powder to deionized water. After being fully stirred for 30 min by a magnetic mixer, the soybean powder solution was left standing in a low temperature environment for 4 h. The static soybean powder solution was centrifuged at 3000 r/min for 15 min, and the supernatant obtained after centrifugation was the urease solution required for the test.

#### 2.2.2. EICP

The cementing solution was a mixture of urea and calcium salts at equal concentrations. An amount of 10 mL urease solution was mixed with an equal volume of cementing solution in a beaker and the mixture was left standing in an incubator at 30 °C. The composition of the calcification test solution is shown in Table 1. The mass of calcium carbonate was determined by acid pickling method. The theoretical mass of calcium carbonate can be obtained by the following formula:

*TM*_c_ = C × V × M (5)

(6) $$Pc_{\;}=\frac{Mc}{TM_{c}}\times 100\%$$

where:

*TM*_c_ denotes thetheoretical maximum mass of CaCO_3_ precipitation (g);

C denotes the concentration of cementing solution (mol/L);

V denotes the volume of solution in the beaker (L);

M denotes the molar mass of CaCO_3_ (100.087 g/mol);

*P*_c_ denotes CaCO_3_ productivity (%);

*M*_c_ denotes the mass of CaCO_3_ precipitation (g).

**Table 1:** Treatment Solution.

Urease Source	Bean Powder Concentration (g/L)	Equimolar Concentration Urea–CaCl_2_ (mol/L)
Soybean	40	0.25
Soybean	40	0.5
Soybean	40	0.75
Soybean	40	1
Soybean	40	1.25
Black bean	40	0.25
Black bean	40	0.5
Black bean	40	0.75
Black bean	40	1
Black bean	40	1.25

#### 2.2.3. Sand Improvement

**Figure 3:**
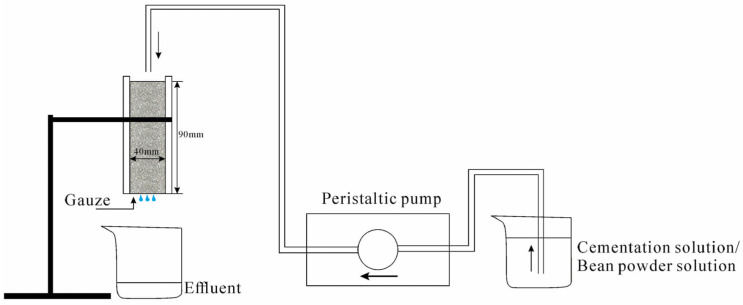
Schematic diagram of sand solidification test.

A PVC pipe with a diameter of 40 mm and a height of 90 mm was used as the mold to prepare the sand column. The bottom of PVC pipe was wrapped with gauze, and the sand soil was put into the PVC pipe after drying at 105 °C. The urease solution of 1.2 times the pore volume of sand was injected with a peristaltic pump at a rate of 4 mL/min. The urease solution was left for 1 h after the injection to fully diffuse into sand particles. An equal volume of cement solution was injected at the same rate once a day for 7 days [27]. The specific process is shown in Figure 3. After being cured, the samples were taken off from PVC pipe.

#### 2.2.4. Unconfined Compressive Strength (UCS) Tests of Improved Sand

The samples were dried with an oven at 105 °C for 24 h. Unconfined compressive strength (UCS) tests at a loading rate of 0.5 mm/min were conducted on the dried samples according to GB/T 50123-2019 [33]. It is important to note that the sample should remain intact before the unconfined compressive strength test

#### 2.2.5. Determination of Calcium Carbonate Content

Gravimetric acid digestion was employed to measure the carbonate content of a portion of each specimen following unconfined compression testing. The damaged sand samples were soaked in hydrochloric acid. A sign that the calcium carbonate has completely reacted is that no bubbles are formed in the hydrochloric acid. The soaked specimens were then rinsed and dried. The mass difference before and after the acid digestion was considered to be the mass of calcium carbonate precipitated in the sample [34].

#### 2.2.6. XRD and SEM

A small sample of sand was collected from three types of sand and was used to examine the effect on morphology and mineralogical composition of the sand due to precipitated minerals using scanning electron microscopy (SEM) and X-ray powder diffraction (XRD) test. Trace sand samples are glued directly to the conductive adhesive and sprayed for 45 s using the Oxford Quorum SC7620 sputtering coating instrument, with a gold spray of 10 mA. The sample topography was then photographed using a TESLA MIRA LMS scanning electron microscope (SEM). The crystal structure of the CaCO_3_ was determined by a powder X-ray diffraction (XRD) with a X’PERT PRO MPD diffractometer using Cu Kα radiation (λ = 0.15406 nm).

## 3. Results and Discussion

### 3.1. Preference of Cementation Solution

**Figure 4:**
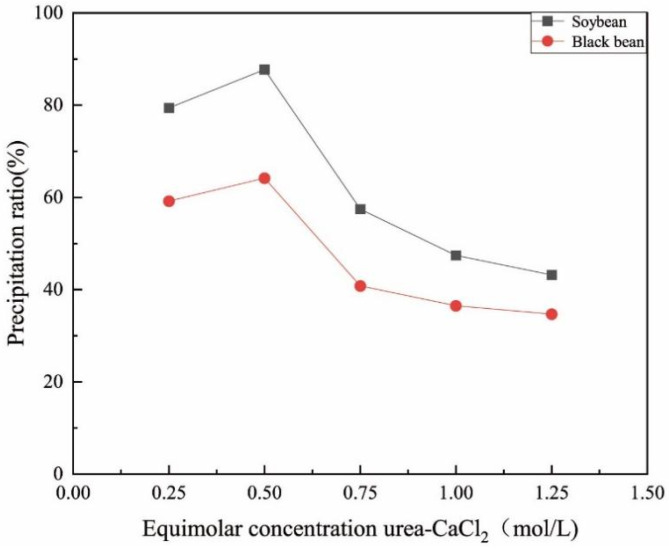
The relationship between the equimolar concentration urea–CaCl_2_ and the precipitation ratio.

Figure 4 shows the difference between soybean urease and black bean urease in promoting the calcium carbonate precipitation. The productivity of calcium carbonate induced by soybean urease was significantly higher than that of black bean urease. The highest precipitation productivity of calcium carbonate induced by soybean urease was 87.7% and that induced by black bean urease was 64.2%.

Figure 4 also reveals the effect of urea–CaCl_2_ cementing solution concentration on the calcium carbonate productivity. Under the action of the two ureases, the calcium carbonate productivity presented the same trend with the change of cementing solution concentration. Calcium carbonate productivity increased with the increase of the cementing solution concentration for low concentrations of cementing solution. However, the increase of cementing solution concentration inhibited the production of calcium carbonate when the concentration of cementing solution exceeds 0.5 mol/L because a high concentration of cementing solution inhibited urease activity [22,35,36]. The highest calcium carbonate productivity was obtained at a cementation solution of 0.5 mol/L concentration.

### 3.2. Effect of Nucleating Agent on Calcium Carbonate Productivity

**Figure 5:**
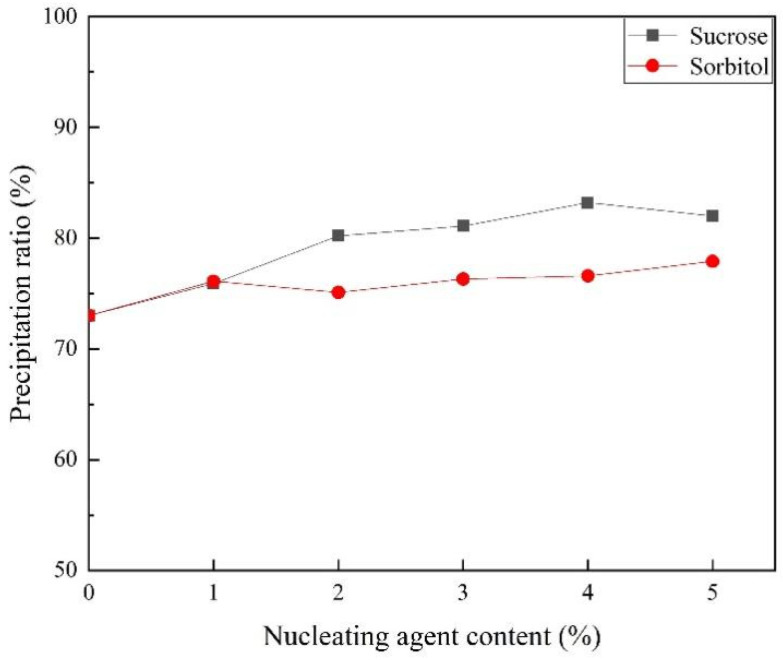
The relationship between nucleating agent content and the precipitation ratio.

EICP provides no nucleation sites for calcium carbonate [37], and the increase in soil strength after EICP solidification is caused by the close point-to-point contact between calcium carbonate and soil particles [38]. In this study, sucrose and sorbitol were added to the cementing solution. Soybean urease and 0.5 mol/L urea–CaCl_2_ cementing solution with the best precipitation effect were selected for the test. Figure 5 shows the effects of two nucleating agents with different amounts on calcium carbonate productivity. The curve revealed nucleating agents had positive effect on the soybean urease precipitation of calcium carbonate. With the increase of nucleating agent, the calcium carbonate productivity showed a slow increase trend. In the experiment of using sorbitol as a nucleating agent, the best effect was obtained when the addition of sorbitol reached 5% and the productivity of calcium carbonate was increased by 5.1%. The addition of sucrose as a nucleating agent to EICP is more effective in increasing the productivity of calcium carbonate. The productivity of calcium carbonate was increased by 12.3% at most when 4% sucrose was added. In this study, 4% is considered to be a suitable maximum addition of a nucleating agent based on economics and improved effects.

### 3.3. Unconfined Compressive Strength

**Figure 6:**
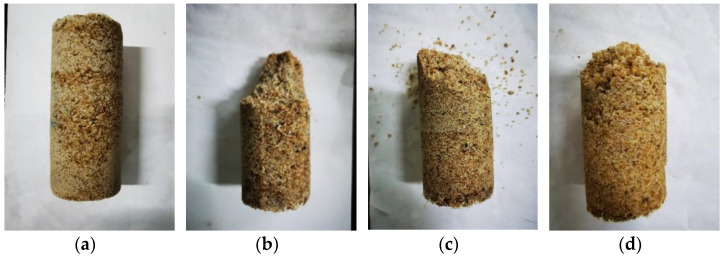
Morphology of sand samples. (**a**) Complete sand column sample. (**b**) Damaged morphology of sand column sample without nucleating agent added. (**c**) Damaged morphology of sand column sample with added sucrose. (**d**) Damaged morphology of sand column sample with added sorbitol.

Due to its simplicity and effectiveness, the unconfined compressive strength (UCS) test is often applied in the evaluation of the effect of EICP treated soil [24,29,39,40]. The integrity of the samples should be ensured before the test. Regarding the residual strength of the damaged sand samples, it was found that the residual strength value was similar to the unconsolidated sand and had no connection with the calcium carbonate content in the samples. This indicated that the strength of the samples almost completely lost once the calcium carbonate cement was broken as a whole [11]. The low residual strength attribute highlighted the importance of careful sample handling prior to testing. As shown in Figure 6, the surface of the samples treated by EICP was smooth and the particles were well cemented with calcium carbonate; the damaged modes of specimens were basically the same after the unconfined compressive strength test.

**Figure 7:**
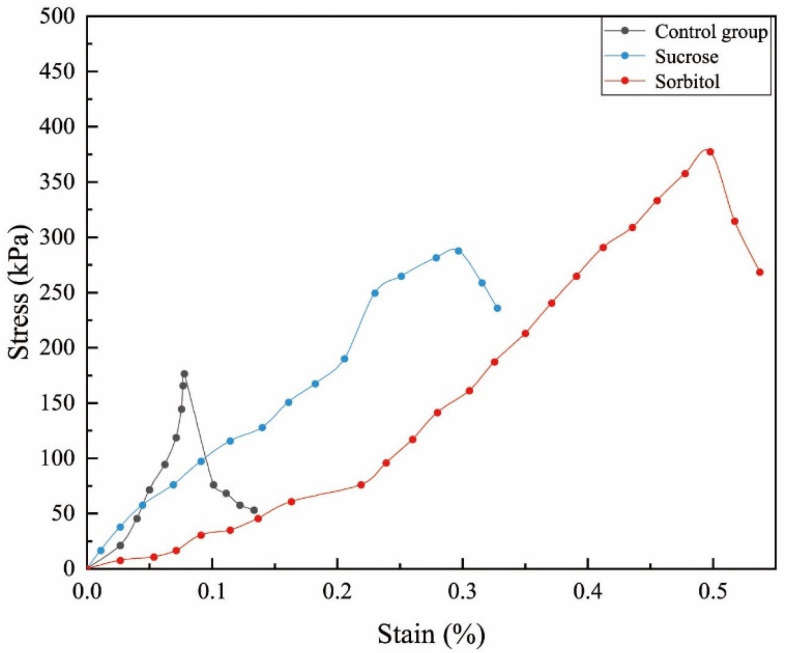
Stress–strain response of EICP solidified sand with different nucleating agents.

Figure 7 presents the stress–strain curve derived from the unconfined compressive strength (UCS) test. The cement solution at a concentration of 0.5 mol/L was used to prepare the sample, and a 4% solution mass of sucrose and sorbitol was added to the cementation solution. Compared to the control group, the stress–strain curves of the samples added with sucrose and sorbitol moved significantly to the upper right, showing that the UCS and strain increased obviously. More calcium carbonate is cemented between soil particles, increasing the contact points between soil particles and causing the stress–strain curve to change. Nucleating agents can be considered effective for improving EICP.

**Figure 8:**
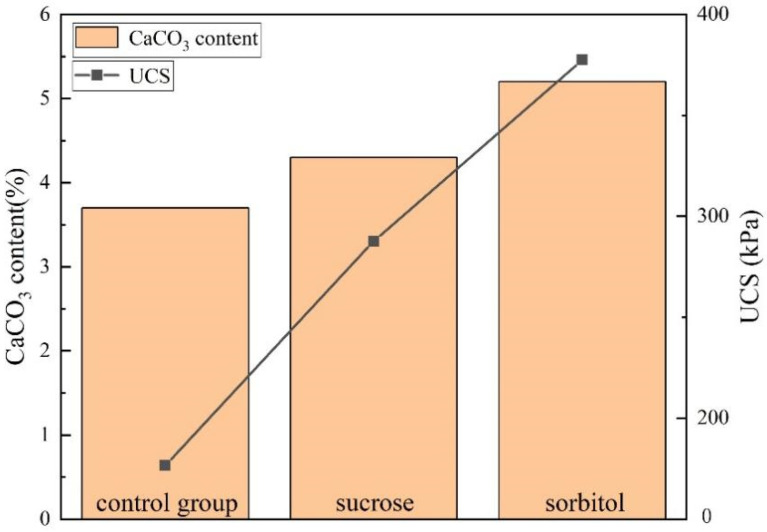
The relationship between CaCO_3_ content and UCS.

A lot of tests have proved that the UCS is closely related to the content of calcium carbonate in soil samples [41]. The calcium carbonate content in the sample is positively correlated with the UCS, and this corresponding relationship varies under different test conditions [31,32,42]. Figure 8 shows the relationship between calcium carbonate content and UCS in this study. When the UCS of the control group was 176.6 kPa, the content of CaCO_3_ in the sample was 3.7%. After sucrose and sorbitol were introduced into EICP. The UCS of the sample increased to 287.7 kPa and 387.5 kPa, respectively, and the CaCO_3_ content in the samples was 4.3% and 5.2%.

### 3.4. Microscopic Mechanisms

**Figure 9:**
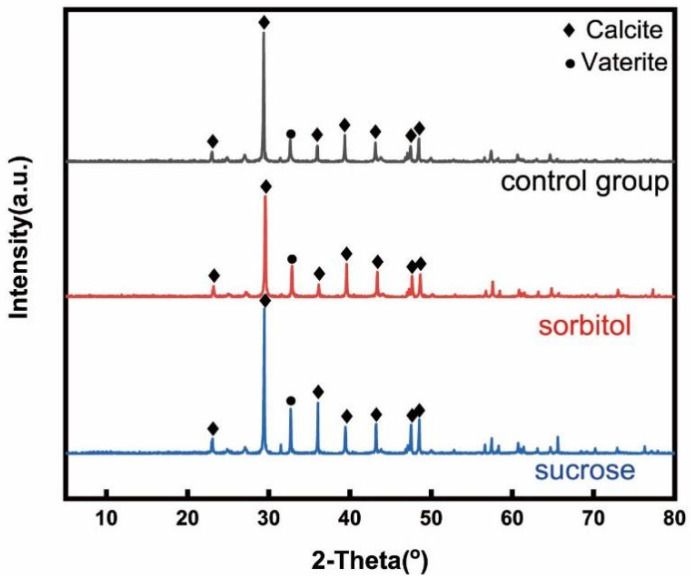
XRD analysis results of CaCO_3_.precipitation.

The XRD patterns of CaCO_3_ in the unmodified EICP, and the EICP-treated sand samples improved by nucleating agents are shown in Figure 9. In all samples, CaCO_3_ mainly existed in the form of calcite and part of CaCO_3_ is vaterite. The same diffraction peak still existed in the samples after sucrose and sorbitol were added, indicating that the nucleating agents failed to promote the phase transformation from vaterite to calcite.

**Figure 10:**
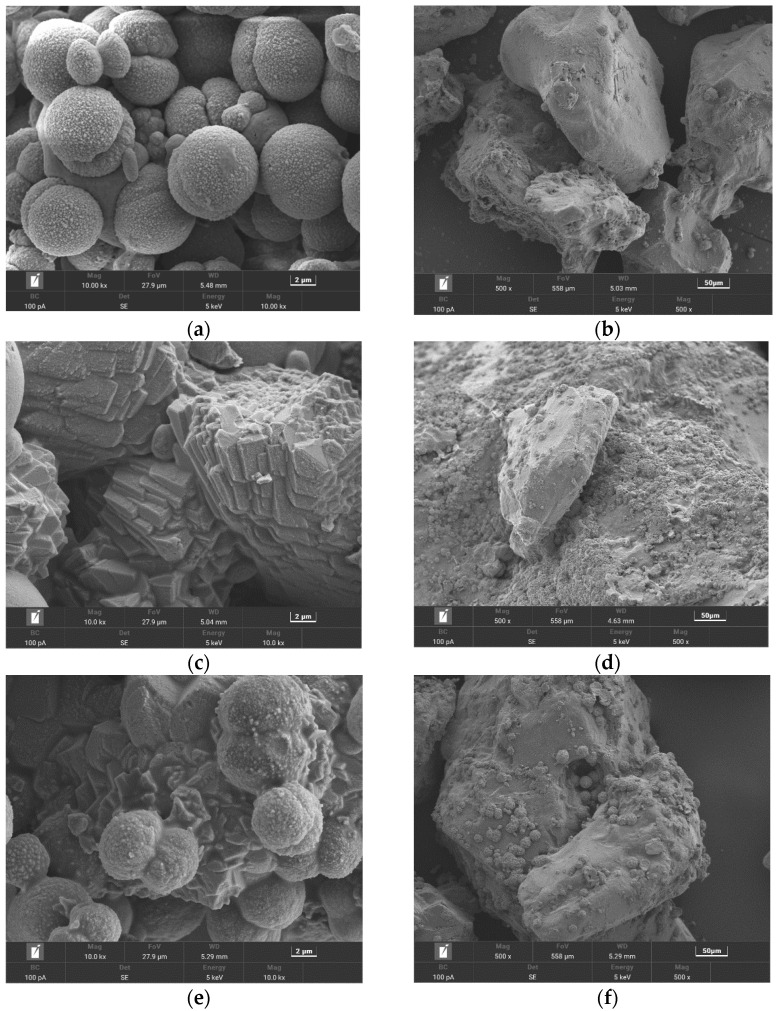
SEM images for sand treated by EICP at (**a**) 10,000× magnification and (**b**) 500× magnification; for sand treated by EICP with sorbitol addition of 4% at (**c**) 10,000× magnification and (**d**) 500× magnification; and for sand treated by EICP with sucrose addition of 4% at (**e**) 10,000× magnification and (**f**) 500× magnification.

Figure 10 shows the SEM images of representative samples. As far as morphology is concerned, EICP mainly produced spherical CaCO_3_ (Figure 10a). After the addition of sorbitol and sucrose to EICP, rhombic and spherical CaCO_3_ aggregated clusters appeared (Figure 10c,e). Although some areas formed relatively concentrated calcium carbonate crystals after being strengthened by EICP, which filled in the pores in the soil particles and cemented the soil particles, many noncontact areas existed between the soil particles (Figure 10b). This is because the urease used in EICP lacks nucleation sites, which had a negative impact on the continuous stacking, bonding and formation of large calcium carbonate [1,43]. In the soil treated by EICP with sorbitol or sucrose, a large amount of CaCO_3_ was produced on the surface of the particles and the pore among adjacent particles (Figure 10d,f). This suggests that the addition of sorbitol and sucrose can compensate for the lack of nucleation sites in EICP.

## 4. Conclusions

In this study, the effect of CaCO_3_ productivity is discussed by changing the urease source in EICP technology and introducing nucleating agents into EICP. At the same concentration of cementing solution, soybean urease has a better effect in calcium carbonate precipitation than black bean. The productivity of calcium carbonate precipitation through urease reaches the maximum value when the concentration of cementing solution is 0.5 mol/L. The unconfined compressive strength of EICP-treated sand is significantly improved after the nucleating agent is improved, and the UCS of the samples is increased by 2.2 times after the sorbitol added. Although sucrose and sorbitol fail to promote the phase transformation of the CaCO_3_ crystal form from vaterite to calcite in the process of EICP, they provide the nucleation sites for CaCO_3_ in EICP, which gives the sand a higher strength after EICP treatment. Another reason for the increase in strength is that sucrose and sorbitol enhance the productivity of CaCO_3_.

## Data Availability

Not applicable.
